# Enrichment of Sialylated IgG by Lectin Fractionation Does Not Enhance the Efficacy of Immunoglobulin G in a Murine Model of Immune Thrombocytopenia

**DOI:** 10.1371/journal.pone.0021246

**Published:** 2011-06-23

**Authors:** Theresa Guhr, Judith Bloem, Ninotska I. L. Derksen, Manfred Wuhrer, Anky H. L. Koenderman, Rob C. Aalberse, Theo Rispens

**Affiliations:** 1 Sanquin Research and Landsteiner Laboratory, Academic Medical Centre, University of Amsterdam, Amsterdam, The Netherlands; 2 Department of Product Development, Sanquin, Amsterdam, The Netherlands; 3 Biomolecular Mass Spectrometry Unit, Department of Parasitology, Leiden University Medical Center, Leiden, The Netherlands; Institut Jacques Monod, France

## Abstract

Intravenous immunoglobulin G (IVIg) is widely used against a range of clinical symptoms. For its use in immune modulating therapies such as treatment of immune thrombocytopenic purpura high doses of IVIg are required. It has been suggested that only a fraction of IVIg causes this anti immune modulating effect. Recent studies indicated that this fraction is the Fc-sialylated IgG fraction. The aim of our study was to determine the efficacy of IVIg enriched for sialylated IgG (IVIg-SA (+)) in a murine model of passive immune thrombocytopenia (PIT). We enriched IVIg for sialylated IgG by S*ambucus nigra agglutinin* (SNA) lectin fractionation and determined the degree of sialylation. Analysis of IVIg-SA (+) using a lectin-based ELISA revealed that we enriched predominantly for Fab-sialylated IgG, whereas we did not find an increase in Fc-sialylated IgG. Mass spectrometric analysis confirmed that Fc sialylation did not change after SNA lectin fractionation. The efficacy of sialylated IgG was measured by administering IVIg or IVIg-SA (+) 24 hours prior to an injection of a rat anti-mouse platelet mAb. We found an 85% decrease in platelet count after injection of an anti-platelet mAb, which was reduced to a 70% decrease by injecting IVIg (p<0.01). In contrast, IVIg-SA (+) had no effect on the platelet count. Serum levels of IVIg and IVIg-SA (+) were similar, ruling out enhanced IgG clearance as a possible explanation. Our results indicate that SNA lectin fractionation is not a suitable method to enrich IVIg for Fc-sialylated IgG. The use of IVIg enriched for Fab-sialylated IgG abolishes the efficacy of IVIg in the murine PIT model.

## Introduction

Intravenous immunoglobulin G (IVIg) is a therapeutic immunoglobulin G preparation derived from pooled plasma of at least 1000 healthy blood donors. It was initially developed as a replacement agent for treating primary and secondary antibody deficiencies. However, since nearly three decades immune modulating therapy of acute and chronic autoimmune diseases became a second major clinical indication for IVIg therapy [1]–[4]. Many patients with acute and chronic autoimmune diseases benefit from IVIg treatment, although its use is not in all cases effective [5]. From clinical studies it is known that IVIg therapy is effective in the treatment of antibody-dependent thrombocytopenia such as idiopathic thrombocytopenic purpura (ITP) [6], [7]. The main protective effect of IVIg in ITP seems to be the inhibition of the Fcγ receptor mediated phagocytosis [8], [9]. By injecting a high dose of IVIg Fcγ receptor-bearing phagocytic cells in the spleen are blocked and thereby prevent the destruction of antibody opsonized platelets. F(ab')_2_ fragments of IVIg are not able to inhibit platelet clearance in a murine model of thrombocytopenia [8].

Besides the Fcγ receptor inhibition, another reason for the high dose (1.0–2.0 g/kg) of IVIg required for therapeutic efficacy could be that only a fraction of IVIg is causing the desired effects. Identification of this fraction of IVIg would possibly allow the development of a more effective IVIg preparation with a changed composition designed for treating patients with autoimmune diseases.

A recent study indicated that the Fc-sialylated IgG fraction is the active immunomodulating entity in IVIg [10]. In this study, the authors enriched IVIg for IgG containing sialic acid (IVIg-SA (+)) using S*ambucus nigra agglutinin* (SNA) lectin fractionation. In a murine K/N serum transfer model for rheumatoid arthritis they found a 10-fold enhancement of the protective effect of IVIg-SA (+). By using Fc fragments instead of intact IgG, they demonstrated that sialylated Fc fragments likewise caused an enhanced protection of the mice similar to IVIg-SA (+). They concluded that the anti-inflammatory activity of IVIg is limited to Fc-sialylated IgG molecules. Two years later the same group confirmed the results by showing that a fully recombinant, sialylated IgG1 Fc domain caused a comparable protective effect [11]. The authors extended these findings by showing that SIGN-R1 is involved in the binding of sialylated Fc fragments [12].

The abovementioned results of enhanced protection by using sialylated Fc fragments are very convincing, although it is debatable whether the used method is suitable to enrich IVIg for Fc-sialylated IgG. A recent study has demonstrated that the binding of IVIg to SNA lectin is primarily mediated by Fab glycosylation, and that for binding of the Fc part to the SNA lectin column two sialic acid residues are required [13]. Analysis of the glycosylation patterns of IVIg revealed that less than 1% of Fc parts contain two sialic acid residues [13]. An earlier study showed that a sialic acid residue attached to Fc part tends to be hidden within the interface between the two CH2 domains which makes this sialic acid residue inaccessible for SNA lectin binding [14]. They demonstrated that under native conditions SNA lectin binding is restricted to the sialic acid residues attached to the Fab part. SNA lectin bound to the Fc part only under reducing conditions (which opens up the interface between the CH2 domains) [14].

The aim of our study was to extend these findings to another case where immune modulating effects of IVIg are most likely Fc-mediated. Therefore, we wanted to investigate whether IVIg enriched for sialylated IgG using SNA lectin fractionation enhances the efficacy (immune modulating effect) of IVIg in a murine model of passively induced immune thrombocytopenia (PIT). Our results indicate that SNA lectin fractionation is not a suitable method to enrich IVIg for Fc-sialylated IgG. Moreover, use of IVIg enriched for Fab-sialylated IgG resulted in a decrease rather than an increase of the efficacy of IVIg in the used murine model of passive immune thrombocytopenia.

## Results

### SNA-lectin affinity-enriched IVIg

**Figure 1:**
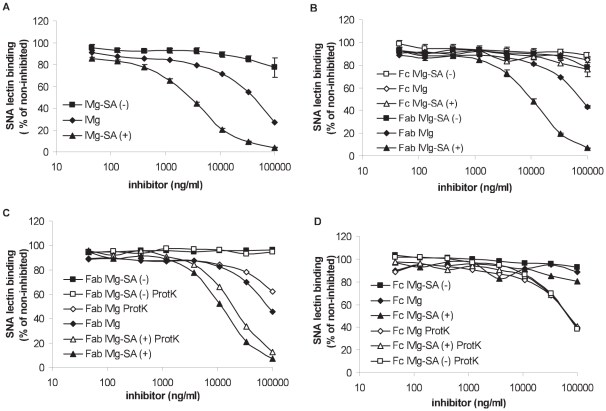
Measurement of the degree of sialylation by SNA lectin inhibition ELISA. % SNA lectin binding after pre-incubation **A**) with IVIg, IVIg-SA (+) or IVIg-SA (−) **B**) with Fab and Fc fragments derived from IVIg, IVIg-SA (+) or IVIg-SA (−) **C**) with Fab or protK digested Fab fragments derived from IVIg, IVIg-SA (+) or IVIg-SA (−) **D**) with Fc or protK digested Fc fragments derived from IVIg, IVIg-SA (+) or IVIg-SA (−). Data represents mean ± SEM, n = 3.

IVIg was enriched or depleted for sialylated IgG by using SNA lectin fractionation as described by Kaneko et al. [10]. This two-step elution resulted in two enriched fractions, with yields of ca. 4.5% and 1.5% for fraction 1 and 2, respectively. The experiments described from here on were carried out using fraction 1 only. However, additional experiments were also carried out using fraction 2, as described in Text S1. Similar results were obtained for both fractions. To measure the degree of sialylation under native (non-reduced) conditions we used a SNA lectin inhibition ELISA. As shown in Figure 1A IVIg-SA (+) inhibited the binding of biotinylated SNA lectin to the IgG coat more efficiently than IVIg, whereas IVIg-SA (−) resulted in a less efficient inhibition. As shown in Table 1, IVIg-SA (+) was approximately 13-fold more potent to inhibit SNA lectin binding to IgG than IVIg, whereas the inhibition by IVIg-SA (−) was minimal and could not be quantified.

**Table 1:** Calculated potencies (depicted as mean with corresponding CI*) of IVIg, IVIg-SA (+), their Fab fragments and Fc protK digests to inhibit SNA lectin binding to IgG.

	IVIg	IVIg-SA(+)	IVIg-SA(−)
**IgG**	1	13 (12.0–14.8)	n.d.
**Fab**	0.49 (0.44–0.54)^a^	3.6 (3.2–4.2)^a^	n.d.
		7.0 (6.0–8.3)^b^	
**Fc protK digest**	0.39 (0.34–0.46)^a^	0.39 (0.33–0.45)^a^	0.41 (0.36–0.46)^a^
		0.87 (0.73–1.0)^c^	0.95 (0.81–1.11)^c^

a ) relative to IVIg-IgG.

b ) relative to IVIg-Fab.

c ) relative to IVIg-Fc protK digest.

*CI confidence interval.

n.d. not determined.

Next, we determined the site of sialylation by measuring the degree of Fab- and Fc-sialylation. Fab fragments derived from IVIg-SA (+) were approximately seven-fold more potent to inhibit SNA lectin binding to IgG than Fab fragments derived from IVIg, whereas no inhibition of SNA lectin binding was visible using Fab fragments derived from IVIg-SA (−) (Figure 1B and Table 1). Importantly, none of the Fc fragments were able to inhibit SNA lectin binding to IgG.

After digestion of the Fc fragments with proteinase K we observed an inhibition of the SNA lectin binding to IgG indicating that under denatured conditions the sialic acid residues attached to the Fc part are available for SNA lectin binding. The inhibition of the SNA lectin binding to IgG was approximately 2.5 fold less potent than IVIg. Moreover, the degree of inhibition was identical for all Fc fragments (Table 1). Similarly, Western blot analysis showed that under reduced conditions SNA lectin binding to the Fc fragments was identical (data not shown) confirming the ELISA results and results of earlier studies [13], [14].

**Figure 2:**
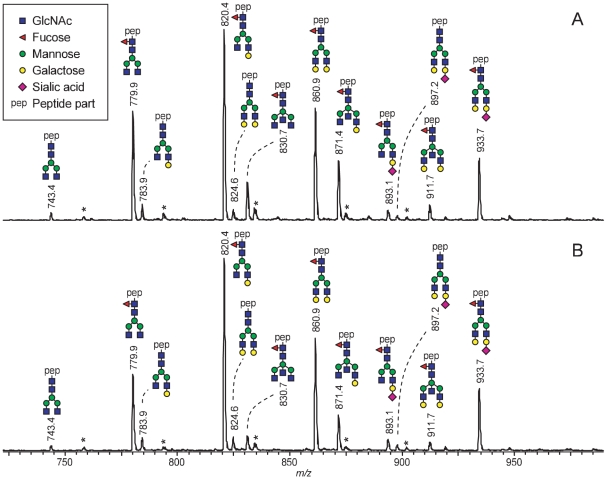
Mass spectrometric IgG1 Fc-glycosylation profiles of A) IVIg and B) IVIg-SA (+). A) IVIg and B) IVIg-SA (+) were subjected to tryptic cleavage and analyzed by nanoLC-ESI-ion trap-mass spectrometry. Sum mass spectra of the elution range of the IgG1 Fc-glycopeptides are shown. Glycopeptides were registered as proton adducts ([M+4H]^4+^). All the displayed glycopeptides have 1 missed tryptic cleavage site and share the peptide moiety T_289_KPREEQFNSTFR_301_ carrying a glycan at N_297_. *, contaminant or irrelevant peak.

To confirm the ELISA results, the degree of Fc sialylation of IVIg and IVIg-SA (+) was determined by mass spectrometric analysis of glycopeptides (Figure 2). Notably, the specific peptide moiety present on the registered glycans ensures that this method only registers IgG1 Fc glycosylation, without any interference from Fab glycosylation [13], [15]. The glycosylation profiles obtained for the two samples were very similar and showed no enrichment of sialylated Fc glycoforms in IVIg-SA (+). Signal intensities were quantitatively compared, and on the basis of the mass spectrometric signals very similar Fc glycopeptide sialylation levels of 12% and 13% were determined for IVIg and IVIg-SA (+), respectively (Table 2). Likewise, galactosylation (55% and 58%) and fucosyolation (94% and 93%) levels were found to be very similar for IVIg and IVIg-SA (+), respectively. A noticable difference was found for the incidence of bisecting GlcNAc, which was 18% and 11% for IVIg and IVIg-SA (+), respectively.

**Table 2:** IgG1 Fc-glycosylation features of IVIg before and after SA-enrichment.

		Incidence (%)	
Glycosylation feature	Registered signal (*m/z*)	IVIg	IVIg-SA (+)
H3N4	743.4	1	1
H3N4F1	779.9	16	15
H4N4	783.9	2	2
H4N4F1	820.4	31	36
H5N4	824.6	2	2
H3N5F1	830.7	6	3
H5N4F1	860.9	18	20
H4N5F1	871.4	10	7
H4N4F1S1	893.1	2	2
H5N4S1	897.2	1	1
H5N5F1	911.7	2	2
H5N4F1S1	933.7	10	10
Galactosylation	-	55	58
Sialylation	-	12	13
Incidence of bisecting GlcNAc	-	18	11
Fucosylation	-	94	93

Glycopeptides were registered by nanoLC-ESI-ion trap-mass spectrometry (see Figure 2). Peak heights of glycopeptide signals (proton adducts [M+4H]^4+^) were integrated and normalized to the total peak heights of glycopeptide signals. The average of two measurements is given.

### Murine model of passive-immune thrombocytopenia (PIT)

#### Platelet count

**Figure 3:**
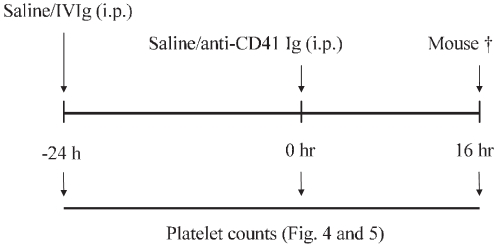
Schematic representation of the passive-immune thrombocytopenia mouse model.

The biological effect of sialylated IgG was tested using a well established murine model of passive-immune thrombocytopenia as described earlier [8] (Figure 3).

**Figure 4:**
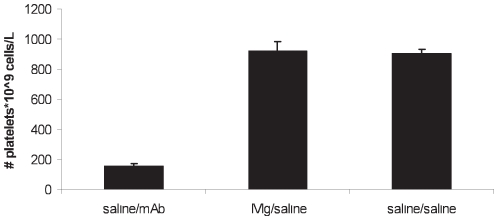
Measured platelet count at t = 16 hours of the control groups. Results are depicted as mean with error bars representing standard error of mean (SEM), (n = 10–12 mice per group). At t = −24 h, platelet counts were 913±52*10^9^, 915±28*10^9^, and 867±38*10^9^ cells/L, respectively.

As shown in Figure 4, mice receiving mAb (saline/mAb group) displayed severe thrombocytopenia. The platelet count dropped to a level of around 15% (154±20*10^9^ cells/L) compared to the control mice 923±61*10^9^ cells/L (saline/saline group). Furthermore, administration of solely IVIg did not by itself cause any changes in platelet count (IVIg/saline group, 905±27*10^9^ cells/L).

**Figure 5:**
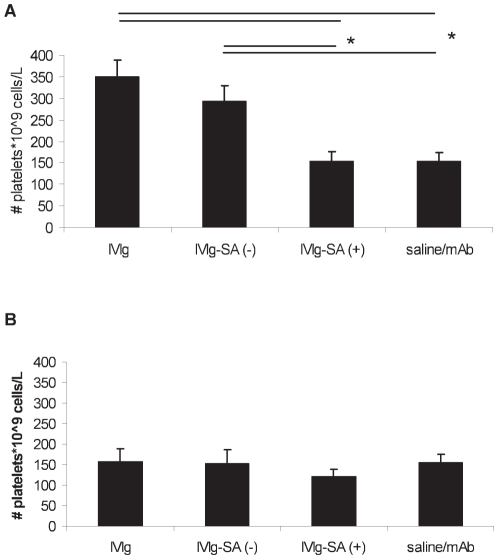
Measured platelet count at t = 16 hours after IVIg pre-treatment. Pre-treatment with **A**) a high dose (1.5 g/kg) of IVIg, IVIg-SA (−), IVIg-SA (+), or saline. **B**) a low dose (0.3 g/kg) of IVIg, IVIg-SA (−) IVIg-SA (+), or saline. Results are depicted as mean with error bars representing standard error of mean (SEM), (n = 10–12 mice per group). * *P*<0.01.

Pre-treatment with a high dose (1.5 g/kg) IVIg had a protective effect (Figure 5A). The mice that received IVIg or IVIg-SA (−) had a significantly higher platelet count (350±39 and 292±36*10^9^ cells/L, respectively) compared to the mice receiving no IVIg pre-treatment (*P*<0.01). Thus, a single dose of IVIg or IVIg-SA (−) resulted in an increase of platelets, reaching a platelet count of about 35% of baseline value. Our results confirm the results of Crow et al. [8] but are in contrast with the results of Teeling et al. who found that the platelet clearance after the induction of thrombocytopenia was only reduced using an IVIg product which underwent a process of ‘ageing’ [16].

In contrast to IVIg and IVIg-SA (−), no increase in platelet count was observed after pre-treatment with IVIg-SA (+). The platelet count dropped to the same level (154±70*10^9^ cells/L) as for the mice receiving no IVIg pre-treatment. Treatment with the lower dose (0.3 g/kg) had no effect (Figure 5B). In separate experiments, also IVIg-SA (+) from fraction 2 was tested and compared to IVIg-SA (+) from fraction 1. Neither of these materials resulted in an increase in platelet count (Figure S1).

#### Human IgG concentration and subclass distributions

To rule out the possibility that the lower efficacy of IVIg-SA (+) was caused by a lower human IgG concentration in the blood circulation of the mice, we determined the human IgG concentration at t = 16 hours using an anti-human IgG ELISA. We observed no difference in human IgG concentrations in mice after injecting a high dose (1.5 g/kg) of IVIg, IVIg-SA (−) or IVIg-SA (+). The human IgG concentration at t = 16 hours in the mice which received IVIg, IVIg-SA (−), IVIg-SA (+) and IVIg without mAb injection was respectively 2.2±0.1, 2.5±0.2, 2.6±0.2 and 2.3±0.2 mg/ml. There was no difference in human IgG concentration after pre-treatment with a low dose (0.3 g/kg) of IVIg (data not shown).

IgG subclass distributions of IVIg-SA (+) and IVIg were also determined (Table S1). The IgG subclass distribution of IVIg-SA (+) was not substantially different compared to IVIg, ruling out the possibility that the lower efficacy of IVIg-SA (+) is caused by an altered IgG subclass distribution.

## Discussion

In the present study we used a murine model of thrombocytopenia to evaluate the effect of IVIg that differed in the amount of sialylated IgG. We demonstrated that enrichment for sialylated IgG did not enhance the efficacy of IVIg. By contrast, the clearance of platelets could only be reduced by administrating IVIg or IVIg-SA (−). IVIg-SA (+) had no effect on the platelet count. This is in contrast to our hypothesis, which was based on the findings of Kaneko et al. [10] and Anthony et al. [11].

We used the same method as described by Kaneko et al. [10], and our ELISA analysis showed that we enriched for sialylated IgG. However, we predominantly found enrichment in Fab-sialylated IgG and not for Fc-sialylated IgG. Further analysis revealed that SNA lectin binding to the Fc part only occurred under non-native conditions (e.g. protK digestion or Western blotting). From the literature it is known that monosialylation of the Fc part is not sufficient for Fc binding to SNA lectin [14], [17], [18]. Thus, our results indicate that IVIg contains predominantly IgG with monosialylated Fc parts which are not available for SNA lectin binding under native conditions and thereby did not result in a substantial increase of Fc-sialylation in IVIg. Nevertheless, it was suggested by Stadlmann et al. that enrichment by SNA lectin chromatography is limited to disialylated Fc parts [13], [14], a fraction too small to detect but arguably responsible for the immune modulating effects of IVIg as found by Kaneko et al. Because we essentially enriched for sialic acid similar to Kaneko et al., we propose that the lack of efficacy of IVIg-SA (+) in our model points to a different mechanism of action of IVIg in the PIT model compared to the murine K/N serum transfer model for rheumatoid arthritis.

Various mechanisms of action of IVIg on different levels of the immune system have been reported [19]–[23]. An important immunomodulating mechanism of IVIg is the immune regulation through the inhibitory Fcγ-receptor (FcγRIIb) on effector macrophages [22], [23]. In ITP, the main protective effect of IVIg on platelet clearance is FcγRIIb-mediated as IVIg had no effect on the platelet count in FcγRIIb ^−/−^ mice [24]–[26]. Our study demonstrates that IVIg-SA (+) is less protective than IVIg on the Fcγ-receptor mediated platelet clearance. This lesser efficacy of IVIg-SA (+) might be caused by a lower binding affinity to the Fcγ-receptors which has been reported previously [10], [12]. Although FcγRIIb is required for IVIg to be protective in the arthritis model, the binding of IVIg-SA (+) to the FcγRs was reduced (10-fold lower affinity) indicating that other receptors are also involved [10], [12]. Further research using isolated Fc parts showed that in mice the SIGN-R1 receptor and in humans DC-SIGN, the human orthologue of SIGN-R1 is required, suggesting a SIGN-R1/DC-SIGN mediated receptor signalling of IVIg-SA (+) [12]. Besides the SIGN-R1/DC-SIGN mediated receptor signalling Séïté et al. [27] have recently demonstrated that IVIg-SA (+) modulates B cell receptor signalling through the binding to CD22 causing B cell apoptosis. Thus, our results together with the literature suggest that enhanced efficacy by treatment with IVIg-SA (+) instead of IVIg requires the involvement of other receptors (e.g. SIGN-R1/DC-SIGN and CD22 receptor) than FcγRIIb. In ITP this is apparently not the case and therefore IVIg-SA (+) has no enhanced efficacy compared to IVIg.

The lack of increased efficacy of IVIg-SA (+) in our model could be due to our failure to enrich for Fc-sialylated IgG, but this does not explain why IVIg-SA (+) is actually less active than the starting material. This suggests another reason for the lower efficacy of IVIg-SA (+) in our model. One possibility is a selective depletion of the effective antibodies. For instance, it has been suggested that anti-idiotype antibodies to autoantibodies directed to glycoprotein IIb/IIIa on platelets are involved in the protective effects of IVIg [32]. Alternatively, an unknown target might be involved. Fab-sialylated IgG antibodies might be less protective compared to non-sialylated or Fc-sialylated IgG antibodies due to the presence of Fab glycans. Fernandez-Cruz et al. reported that Käsermann and his group found that the distribution of specific antibodies in IVIg after SNA lectin fractionation differed [28] from unfractionated IVIg, but in the scope of this study we did not further analysed whether a selective depletion of effective antibodies occurred. However, we did test if the enriched material has a different IgG subclass distribution, which was not the case, ruling out a selective depletion for one IgG subclass as a cause for the reduced efficacy of IVIg-SA (+).

To exclude that the lower efficacy of IVIg-SA (+) was caused by a lower human IgG concentration in the blood stream of the mice due to a hypothetical shortened half-life of sialylated IgG we measured the human IgG concentration in the blood samples. Our results confirmed the results of Kaneko et al. [10] that the degree of sialylation has no influence on the clearance.

We conclude that SNA lectin fractionation is not a suitable method to enrich IVIg specific for Fc-sialylated IgG. By using this method IVIg is predominantly enriched for Fab-sialylated IgG, which does not enhance the efficacy of IVIg in our murine model of thrombocytopenia. Having our SNA lectin fractionation results and the literature [13], [14], [29] in mind, it is not clear whether the described anti-inflammatory effect of IVIg in the murine K/N serum transfer model for rheumatoid arthritis [10]–[12] can be limited to Fc-sialylated IgG. As IVIg also contains Fab-sialylated IgG [30] the anti-inflammatory activiy of IVIg-SA (+) might be caused also by Fab-sialylated IgG.

Considering the results of this study, further research is required to elucidate the role of IgG sialylation as well as IgG glycosylation in more detail. It would be interesting to study a) the biological effect of IgG containing no, one or two sialic acid residues at the Fc part and IgG with or without a Fab-sialylated part using different murine models (e.g. rheumatoid arthritis and thrombocytopenia and b) the SIGN-R1/DC-SIGN binding of sialylated IgG with and without removal of the Fab- and Fc-linked glycans. It seems that the enhanced efficacy of IVIg-SA (+) is not a general property but depends on the autoimmune disease.

## Materials and Methods

### Animals/Ethics statement

Virgin female Balb/c mice (6 weeks of age) were purchased from Harlan (Harlan CPB, Zeist, Netherlands) and housed in the animal facilities of the Dutch cancer institute. The institutional ethics committee of the Dutch cancer institute approved the animal experiments used in this study, permit number 2008.001.

### Reagents

From Vector Laboratories (Burlingame, USA) we obtained both the biotinylated and the agarose-coupled *Sambucus nigra* agglutinin (SNA) lectin which recognizes α-2,6 linked sialic acid residues (with a binding capacity of 1.5 mg fetuin/ml gel).

To induce thrombocytopenia, we used a rat anti-mouse platelet mAb (MWReg30, IgG1) which targets the platelet-specific integrin α_IIb_β_3_ (gpIIb/IIIa) (Pharmingen, San Diego, CA, USA).

### Immunoglobulin preparations

Human γ-globulin for intravenous (IVIg) use was obtained from Sanquin (Nanogam, 5%; Amsterdam, The Netherlands). This liquid IVIg product is prepared from pooled plasma from at least 1000 healthy donors by Cohn fractionation followed by a low-dose pepsin treatment at pH 4.0, 15 nm filtration, solvent-detergent treatment and formulated as 5% (w/v) solution in 5% glucose solution pH 4.5. It was stored at 4°C.

### Preparation of sialic acid enriched or depleted IVIg by SNA lectin fractionation

To increase the proportion of sialylated IgG (SA-IgG) in IVIg SNA lectin fractionation (also referred as SNA lectin affinity chromatography) was used as described by Kaneko and co-workers [10]. Briefly, IVIg was dialyzed against Tris-buffered saline (TBS) containing 0.1 mM CaCl_2_ and 200 mg dialyzed IVIg was applied to a 10 ml agarose-bound SNA lectin column. The flow-through fraction (SA-IgG depleted IVIg = IVIg-SA (−)) was collected by washing the column with 20 ml TBS containing 0.1 mM CaCl_2_. Next, the sialic acid enriched fraction of IVIg was eluted in 2 steps using 20 ml of 0.5 M lactose in TBS (fraction 1, yield 4.5% followed by 20 ml of 0.5 M lactose in 0.2 M acetic acid (fraction 2, yield 1.5%). All fractions were dialyzed against water and concentrated to a protein concentration of 50 mg/ml using a Centrifugal filter device (cut off 10 kDa, Amicon, Millipore, Billerica, MA, USA). After a sterile filtration, glucose was added to obtain the same formulation as IVIg with a final pH of 4.5.

### Papain digestion of IVIg

To determine the site of sialylation, Fab and Fc fragments were prepared by treating IVIg, IVIg-SA (+) and IVIg-SA (−) with DTT pre-activated papain (Sigma-Aldrich, St. Louis, MO, USA) at an enzyme : antibody ratio of 1∶400 in phosphate buffered saline (PBS) pH 7.4 with 3 mM EDTA at 37°C for 3 hours. The reaction was stopped by adding fresh iodoacetamide (Sigma-Aldrich, St. Louis, MO, USA) to a final concentration of 83 mM. The digested fraction was separated from the undigested fraction by size exclusion chromatography using a Hiload 16/60 Superdex200 column (60 cm, 124 ml, GE Healthcare, Uppsala, Sweden) connected to a HPLC system (GE Healthcare). The Fab fragments were separated from the Fc fragments by charge using a MonoQ column (GE Healthcare). To further purify the Fc fragments protein G affinity chromatography was used. Briefly, we respectively loaded 0.8 mg, 0.7 mg or 1 mg of Fc fragments derived from IVIg-SA (+), IVIg-SA (−) or IVIg on a protein G 4 Fast Flow column with a packed column volume of 250 µl (GE Healthcare). After washing with 10 column volumes PBS the bound Fc fragments were eluted with 6 column volumes 100 mM Glycine pH 2.8 followed by an immediate neutralization with 2 M K_2_HPO_4_. After overnight dialysis against PBS at 4°C and the addition of sodium azide (NaN_3_) with a final concentration of 0.05% the fragments were stored at 4°C.

### Proteinase K digestion of Fc fragments

To determine the degree of Fc sialylation, Fc fragments derived from IVIg, IVIg-SA (+) or IVIg-SA (−) were digested with protK (proteinase K, Sigma-Aldrich, St. Louis, MO, USA). The Fc fragments were overnight dialyzed against PBS at 4°C, followed by incubation with protK (ratio 0.6 U/mg protein, overnight, at 50°C). The protK was inactivated by incubating the mix at 80°C for 3 minutes. After the addition of sodium azide (NaN_3_) with a final concentration of 0.05% the digested Fc fragments were stored at 4°C.

### Determination of the degree of sialylation

To determine the degree of IgG sialylation the following methods were used:

#### Lectin inhibition ELISA

To measure the sialic acid content under native conditions and in protK digests, we used an enzyme-linked immunosorbent assay (ELISA). Biotinylated SNA lectin diluted to 0.5 µg/ml in phosphate buffered saline (PBS) containing 0.1% Tween20 was pre-incubated with the inhibitors (IVIg, IVIg-SA (+), IVIg-SA (−), Fab or Fc fragments) for 1 hour at RT. After adding the mix to an IVIg coated microtiter plate (Maxisorp; Nunc, Roskilde, Denmark), we incubated the plate for 1 hour at RT. Subsequently, the plates were washed and streptavidin-HRP (Amersham, Uppsala, Sweden) diluted 1∶4000 in PBS/0.1% Tween20 was added. After 1 hour incubation at RT the plates were washed and 100 µl/well TMB substrate buffer (Interchim, Montluçon Cedex, France) diluted with an equal volume of distilled water was added. The reaction was stopped with 2 M H_2_SO_4_ after 5 minutes and the absorbance was measured at 450 nm with a Titertek multiscan.

#### Mass spectrometric analysis of IgG1 Fc glycosylation

For mass spectrometric Fc-glycosylation analysis IVIg samples were subjected to trypsin treatment, and resulting glycopeptides were analyzed by nano-LC-ion trap-MS as described elsewhere [15]. In accordance with an earlier report IgG1 Fc-glycopeptides exhibited a missed tryptic cleavage site resulting in the peptide moiety T_289_KPREEQFNSTFR_301_ carrying a glycan at N_297_ [17]. Glycopeptides were assigned in terms of monosaccharide composition, i.e. hexose (H), N-acetylhexosamine (N), deoxyhexose (F), and N-acetylneuraminic acid (S). Peak heights of glycopeptide signals (proton adducts [M+4 protons]^4+^) were integrated and normalized to the total peak heights of glycopeptide signals. Glycosylation features were calculated as follows: galactosylation = H5N4+H5N4F1+H5N4S1+H5N5F1+H5N4F1S1+0.5 * (H4N4+H4N4F1+H4N5F1+H4N4F1S1); sialylation = H4N4F1S1+H5N4S1+H5N4F1S1; incidence of bisecting GlcNAc = H3N5F1+H4N5F1+H5N5F1; fucosylation = H3N4F1+H4N4F1+H3N5F1+H5N4F1+H4N5F1+H4N4F1S1+H5N5F1+H5N4F1S1.

### IgG subclass distribution

IgG subclasses of IVIg, IVIg-SA (+), and IVIg-SA (−) were determined using a nephelometric assay as described previously [31].

### Induction of passive-immune thrombocytopenia (PIT)

To induce passive-immune thrombocytopenia mice were injected intraperitoneally with 2 µg of a rat-anti-mouse CD41 monoclonal antibody (MWReg30, BD Pharmingen, San Diego, CA, USA) in 200 µl PBS containing 0.01% human serum albumin. We measured the effect of sialic acid on IVIg by giving IVIg, IVIg-SA (+), IVIg-SA (−) (1.5 or 0.3 g/kg) or saline intraperitoneally 24 hours prior to injection of MWReg30. From the retro-orbital plexus of anesthetized mice blood samples were collected using tubes containing sodium citrate as anti-coagulant, before IVIg or saline treatment (t = −24), 5 minutes prior to injection of MWReg30 (t = 0) and 16 hours after injection of MWReg30 (t = +16). Within 1 hour of blood collection, the blood samples were diluted 1∶5 in PBS and the number of platelets (PLT) was counted by using the ADVIA 2120 haematology system (Siemens Medical Solutions Diagnostics, Erlangen, Germany).

### Detection of human IgG in mice

To measure the human IgG content in mice, we used an enzyme linked immunosorbent assay (ELISA). A mouse anti-human IgG (MH16-1, Sanquin, Amsterdam, Netherlands) coated microtiter plate (Maxisorp, Nunc, Roskilde, DK) was incubated with mouse serum diluted 1∶100 in 0.9% NaCl/10 mM EDTA. After 1 hour incubation at RT the plates were washed five times with PBS/0.1% Tween20 and 100 µl/well of a polyclonal rabbit anti-human IgG labelled with HRP (Sanquin, Amsterdam, Netherlands) at a concentration of 0.5 µg/ml was added to the wells and incubated 1 hour at RT. The binding was measured adding 100 µl/well of a TMB substrate buffer (Interchim, Montluçon Cedex, France) diluted 1∶2 with distilled water. The human IgG concentration was determined by measuring the absorbance at 450 nm using a Titertek multiscan.

### Detection of MWReg30 in mice

Success of the injection of the rat-anti-mouse CD41 monoclonal antibody (MWReg30) was assessed by measuring the HSA content in mice, which was co-injected with the anti-CD41 antibody. To detect HSA a radioimmunoassay (RIA) was set up. 200 µl sheep-anti-HSA antibody (kindly provided by Sanquin Diagnostics, Amsterdam, Netherlands) was coupled to 200 mg Sepharose according to the manufacturer's instructions (GE Healthcare, Uppsala, Sweden) followed by a dilution in PBS to a concentration of 2 mg/ml. For testing, 50 µl mouse serum was incubated with 250 µl of Sepharose. After rotating overnight at RT, the Sepharose was washed five times with PBS/0.1% Tween and incubated with ^125^I (GE Healthcare, Uppsala, Sweden) radioactive labelled double depleted sheep-anti-HSA antibody (depleted for sheep anti-human IgG antibodies and sheep anti-mouse serum). After overnight incubation at RT the Sepharose mix was washed four times with PBS/0.1% Tween and the radioactivity was measured using a Wallac counter. The sera of saline-injected mice were used as negative control.

### Analysis of data

#### Degree of sialylation

To compare the degree of sialylation of IVIg, IVIg-SA (+), IVIg-SA (−) and their Fab fragments their relative potencies in the SNA lectin inhibition ELISA were calculated by parallel line analysis [29]. A value above 1 for a fraction indicates that this fraction is more potent than IVIg, thus is enriched for sialic acid.

#### PIT mouse model

To analyze differences in platelet count a Student t-test was performed. Two-sided tests with *P*-values less than 0.05 were considered to indicate a statistically significant difference.

Mice were excluded if results of the RIA (see above) was below the cut-off value of the test, indicating an incorrect intraperitoneally injection of the MWReg30 antibody. As cut-off value, the mean binding of the negative control group +2*standard deviation was used. We excluded 6 from the 120 mice from analysis. These mice were randomly distributed along the 10 groups.

## Supporting Information

Figure S1 **Measured platelet count at t = 16 hours after high dose IVIg pre-treatment.** Pre-treatment with a high dose (1.5 g/kg) of IVIg, IVIg-SA (+) 1, IVIg-SA (+) 2, or Saline as described in the text below. Results are depicted as mean with error bars representing standard error of mean (SEM), (n = 9). ** P<0.01, * P<0.05.
(PDF)

Table S1 **IgG subclass distribution in IVIg fractions (in %).**
(PDF)

Text S1 **Platelet counts after pre-treatment with fractions 1 and 2.**
(PDF)
