# Supplementary material for: Enrichment of Sialylated IgG by Lectin Fractionation Does Not Enhance the Efficacy of Immunoglobulin G in a Murine Model of Immune Thrombocytopenia
Source: PLoS One. 2011 Jun 23;6(6):e21246. doi: 10.1371/journal.pone.0021246 (PMC3121734; doi:10.1371/journal.pone.0021246)
Supplement: Figure S1 — Measured platelet count at t = 16 hours after high dose IVIg pre-treatment. Pre-treatment with a high dose (1.5 g/kg) of IVIg, IVIg-SA (+) 1, IVIg-SA (+) 2, or Saline as described in the text below. Results are depicted as mean with error bars representing standard error of mean (SEM), (n = 9). ** P<0.01, * P<0.05. (PDF) [file pone.0021246.s001.pdf]

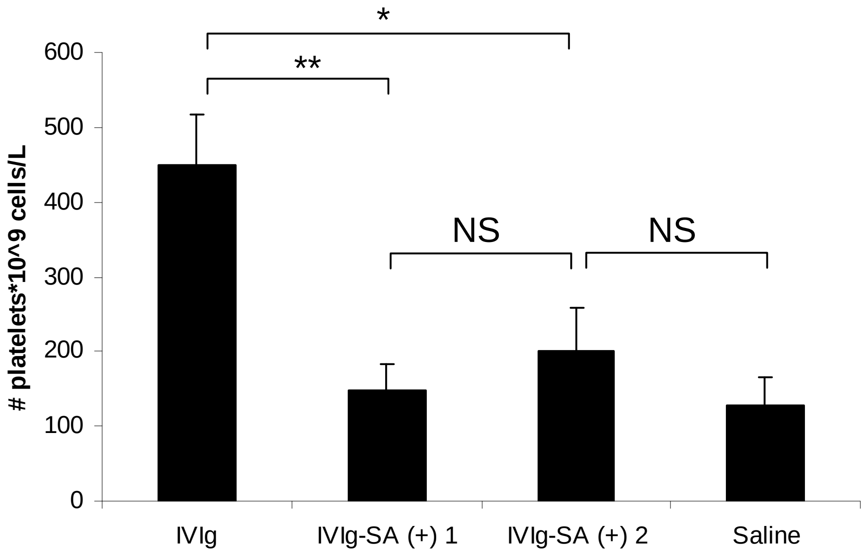

Figure S1. **Measured platelet count at t=16 hours after high dose IVIg pre-treatment.** Pre-treatment with a high dose (1.5 g/kg) of IVIg, IVIg-SA (+) 1, IVIg-SA (+) 2, or Saline as described in the text below. Results are depicted as mean with error bars representing standard error of mean (SEM), (n = 9). ** $P < 0.01$, * $P < 0.05$.
