# Supplementary material for: Enrichment of Sialylated IgG by Lectin Fractionation Does Not Enhance the Efficacy of Immunoglobulin G in a Murine Model of Immune Thrombocytopenia
Source: PLoS One. 2011 Jun 23;6(6):e21246. doi: 10.1371/journal.pone.0021246 (PMC3121734; doi:10.1371/journal.pone.0021246)
Supplement: Table S1 — IgG subclass distribution in IVIg fractions (in %). (PDF) [file pone.0021246.s002.pdf]

Table S1. IgG subclass distribution in IVIg fractions (in %).

	IVIg	IVIg SA(-)	IVIg SA(+)
IgG1	62	62.5	70
IgG2	34	35.3	27
IgG3	3.4	1.8	1.9
IgG4	0.6	0.4	1.1
