# Supplementary material for: Enrichment of Sialylated IgG by Lectin Fractionation Does Not Enhance the Efficacy of Immunoglobulin G in a Murine Model of Immune Thrombocytopenia
Source: PLoS One. 2011 Jun 23;6(6):e21246. doi: 10.1371/journal.pone.0021246 (PMC3121734; doi:10.1371/journal.pone.0021246)
Supplement: Text S1 — Platelet counts after pre-treatment with fractions 1 and 2. (PDF) [file pone.0021246.s003.pdf]

In separate experiments, we carried out the purification as indicated by Kaneko et al. as described in the Materials and Methods Section of the manuscript. However, instead of pooling both fractions after the two-step elution (step 1 lactose; step 2 lactose/acetic acid), we tested both fractions separately for their efficacy in the PIT mouse model. The results are shown in Figure S1. Neither IVIg-SA (+) from fraction 1 nor from fraction 2 were found to be able to diminish the reduction in platelet count induced by the anti-platelet antibody. On the other hand, IVIg was able to partially restore the platelet count to $450\pm 70*10^9$ cells/L. Mean platelet counts before injecting the anti-platelet antibody were $960\pm 40*10^9$ cells/L. The IVIg used in this experiment was from a different lot than the enriched fractions, but both lots were found to contain identical amounts of sialic acid (not shown).
